# Protein Hydrolysates Are Avoided by Herbivores but Not by Omnivores in Two-Choice Preference Tests

**DOI:** 10.1371/journal.pone.0004126

**Published:** 2009-01-05

**Authors:** Kristin L. Field, Alexander A. Bachmanov, Julie A. Mennella, Gary K. Beauchamp, Bruce A. Kimball

**Affiliations:** 1 Monell Chemical Senses Center, Philadelphia, Pennsylvania, United States of America; 2 USDA-APHIS, National Wildlife Research Center, Ft. Collins, Colorado, United States of America; AgroParisTech, France

## Abstract

**Background:**

The negative sensory properties of casein hydrolysates (HC) often limit their usage in products intended for human consumption, despite HC being nutritious and having many functional benefits. Recent, but taxonomically limited, evidence suggests that other animals also avoid consuming HC when alternatives exist.

**Methodology/Principal Findings:**

We evaluated ingestive responses of five herbivorous species (guinea pig, mountain beaver, gopher, vole, and rabbit) and five omnivorous species (rat, coyote, house mouse, white-footed mouse, and deer mouse; N = 16–18/species) using solid foods containing 20% HC in a series of two-choice preference tests that used a non-protein, cellulose-based alternative. Individuals were also tested with collagen hydrolysate (gelatin; GE) to determine whether it would induce similar ingestive responses to those induced by HC. Despite HC and GE having very different nutritional and sensory qualities, both hydrolysates produced similar preference score patterns. We found that the herbivores generally avoided the hydrolysates while the omnivores consumed them at similar levels to the cellulose diet or, more rarely, preferred them (HC by the white-footed mouse; GE by the rat). Follow-up preference tests pairing HC and the nutritionally equivalent intact casein (C) were performed on the three mouse species and the guinea pigs. For the mice, mean HC preference scores were lower in the HC v C compared to the HC v Cel tests, indicating that HC's sensory qualities negatively affected its consumption. However, responses were species-specific. For the guinea pigs, repeated exposure to HC or C (4.7-h sessions; N = 10) were found to increase subsequent HC preference scores in an HC v C preference test, which was interpreted in the light of conservative foraging strategies thought to typify herbivores.

**Conclusions/Significance:**

This is the first empirical study of dietary niche-related taxonomic differences in ingestive responses to protein hydrolysates using multiple species under comparable conditions. Our results provide a basis for future work in sensory, physiological, and behavioral mechanisms of hydrolysate avoidance and on the potential use of hydrolysates for pest management.

## Introduction

Enzymatic hydrolysis of food proteins results in a mixture of peptides and amino acids that are valued for their increased solubility over intact proteins. Hydrolysates generally retain, if not increase, the nutritional and functional properties of the parent proteins. Casein, a protein in milk, is one of the most commonly hydrolyzed proteins for a variety of reasons: its high nutritional quality, the numerous bioactive peptides that have been identified from casein's structure that can be released upon hydrolysis [reviewed in 1], [2], [3], the need for milk-based infant formulas that are hypoallergenic [4], and because humans have a long history with dairy products, such as cheese or fermented drinks, for which hydrolysis is an integral part of the production process [5]. However, the negative sensory properties of casein hydrolysates [e.g., 6], [7], [8] often limit their usage, which has stimulated a large body of research focused on identifying the offensive bitter peptides and on methods for improving the flavor [e.g., 9], [10], [11]. These studies on sensory and nutritive properties of hydrolyzed casein have overwhelmingly been focused on human subjects.

Food containing casein hydrolysate is unappealing to some mammals in addition to humans. Deer (*Odocoileus spp.*) strongly avoided both natural forage and mixed diets that had been adulterated with hydrolyzed casein (HC) [12], [13], and two species of non-ruminant herbivore pests showed depressed intakes when presented diets containing HC [14]. Deer are the only herbivores that have been tested with HC in a choice situation, and it is unknown whether a characteristic of herbivory is avoidance of this protein source when alternatives are available.

The few other studies that have examined responses to HC in which animals were free to choose between an HC-containing food and at least one other alternative, suggest that there is species variability in response to HC. The rat (*Rattus norvegicus*) strongly avoided HC when simultaneously offered three diets containing approximately 20% of either HC, intact casein, or an amino acid mixture simulating casein's amino acid profile source [15]. Lab mice (*Mus musculus*) showed no preference between an HC-containing diet and an intact casein-containing diet over low to moderate protein levels (5–20%). However, when dietary protein concentrations increased (30–50%), these mice avoided the HC diet and selected the casein diet [16]. Domestic and wild cats (*Felis catus* and *Panthera spp.*) preferred an HC solution over water [17], although the maximum concentration (3% HC solutions, *w/v*) used for the feline testing was lower than the 8% concentration of HC solution that successfully minimized deer consumption of tree saplings [13]. However, the variety of concentrations, types of HC, and different matrices in which the HC was presented in these studies prevents even a basic understanding of the nature of this variability.

The study described herein was partially stimulated by HC's potential as a wildlife management tool for protecting agricultural resources. We wanted to determine if herbivores other than deer would avoid this protein source when also given a non-HC containing alternative. Based on the rodent [15], [16] and feline [17] studies, we speculated that there may be differences between herbivores and trophic groups that incorporate animal products in their diets. However, the few species represented and the disparate methodologies of these previous studies make it impossible to determine whether differences would be observed among trophic groups tested under more similar conditions. Thus, this work was also designed to contribute to an initial understanding of the breadth of taxonomic variability in ingestive responses to a complex stimulus that provides a high quality source of protein.

Despite the size and productivity of research areas related to how animals select their diets [e.g., 18], [19], [20], there is still much to learn about how animals respond to potential foods. Empirical work that identifies taxonomic variability in ingestive behavior provides a foundation for directing and/or complementing molecular, physiological and other approaches that seek to understand the mechanisms of this behavior. For diet selection, it is well known that sensory factors and post-ingestive feedback both affect feeding decisions [19]–[21]. An integrative approach that determines whether a dietary stimulus provokes a range of behavioral responses of any interest is a logical initial step when examining novel types of stimuli, which could then be used to direct reductionist approaches towards understanding the involved mechanisms, including the sensory and post-ingestive components of the feeding behavior.

We evaluated ingestive responses of five herbivorous species and five omnivorous species (Table 1) using solid foods containing 20% HC. Animals were either wild-caught or captive-born and could be considered pest species and/or laboratory model species. Most animals were representatives of the order Rodentia, in which we included guinea pigs. Although molecular evidence suggests guinea pigs should be in a unique order [22], none has been designated for them. Lagomorpha and Carnivora were also represented by rabbits and coyotes, respectively. In a series of experiments, individuals of each species were given two-choice preference tests that compared their consumption of an HC-containing diet and an alternative in which the hydrolyzed protein fraction of the diet was replaced with cellulose (Cel). Cel, a plant-based polysaccharide, has no or minimal nutritive value for most mammals, although some species, mostly herbivores, are able to extract some energy from it [23]–[27].

**Table 1:** Species tested in two-choice hydrolysate preference tests, including the short names used for figures and remaining tables.

Scientific name	Common name (short name)	Order	Family	Diet+
*Aplodontia rufa*	mountain beaver (mtnbeaver)	Rodentia	Aplodontiidae	herbivore
*Canis latrans*	coyote (coyote)	Carnivora	Canidae	omnivore
*Cavia porcellus*	guinea pig (g. pig)	Rodentia*	Caviidae	herbivore
*Microtus townsendii*	Townsend's vole (vole)	Rodentia	Cricetidae	herbivore
*Mus musculus*	house mouse (mouse)	Rodentia	Muridae	omnivore
*Oryctolagus cuniculus*	European rabbit (rabbit)	Lagomorpha	Leporidae	herbivore
*Peromyscus leucopus*	white-footed mouse (wfmouse)	Rodentia	Cricetidae	omnivore
*P. maniculatus*	deer mouse (dmouse)	Rodentia	Cricetidae	omnivore
*Rattus norvegicus*	Norway rat (rat)	Rodentia	Muridae	omnivore
*Thomomys mazama*	western pocket gopher (gopher)	Rodentia	Geomyidae	herbivore

* Order is contested; molecular evidence suggests that guinea pigs should be put in their own, unique order [22].

+ See Supporting Information Appendix S1 for the basis for these dietary categorizations.

In a second series of tests, these same individuals were then given two-choice tests between hydrolyzed collagen (gelatin; GE) and the Cel diet. GE is a poor quality protein deficient in essential amino acids [28] that rats have been shown to reject when it is their only protein source [29], [30]. GE's sensory profile is, however, inoffensive (to human palates) [10], [31]. Pilot work showed mice rejected GE more strongly than HC, yet it was the least successful protein for deterring deer from consuming plants when proteins were applied to the plant surfaces [32]. Thus, we included GE in the study to explore whether the individuals tested with HC would respond similarly to another hydrolysate, despite it having little in common with HC except that the native protein had undergone hydrolysis. Our underlying objective was to survey multiple species with the same hydrolysates under similar conditions in order to explore the nature of any variability in feeding responses to a novel protein source. With the guinea pig and three mice species, we performed two follow-up experiments in which HC was paired with an intact casein diet. This pairing eliminated protein content differences between the choices and allowed for some insight into underlying mechanisms affecting consumption of HC.

Thus, our goals in this study for HC were to determine if herbivores other than deer avoid HC-containing food when an alternative is available, to compare herbivore HC responses with those of omnivores, and to identify any pest species for which HC may be effective as a repellent and/or any laboratory species that may provide a good model for future investigation of the mechanisms of HC avoidance. We hypothesized that if any species differences in HC avoidance emerged, they would be related to dietary niche. In general, herbivores use food sources that provide relatively little protein, which is often well protected by the plant, while omnivores and carnivores use relatively high protein foods, which are difficult to locate and/or to capture [33]. Omnivores tend to require higher protein levels from their diet compared to herbivores, which often have mechanisms to efficiently recycle and use nitrogen produced by their microbial symbionts in the forestomach or gut [34]. We expected that these basic differences in protein requirements and typical forage properties would make omnivores more willing to consume unfamiliar protein sources than herbivores, even when the alternative choice was protein-deficient. Our data were broadly consistent with this expectation. For GE, we were interested in whether another hydrolysate would induce similar ingestive responses to those of the HC, despite large differences between the two hydrolysates on nutritional and sensory dimensions. We found that GE response patterns reflected those found for HC.

## Results

### Mean hydrolyzed casein (HC) and gelatin (GE, hydrolyzed collagen) preference scores

**Figure 1:**
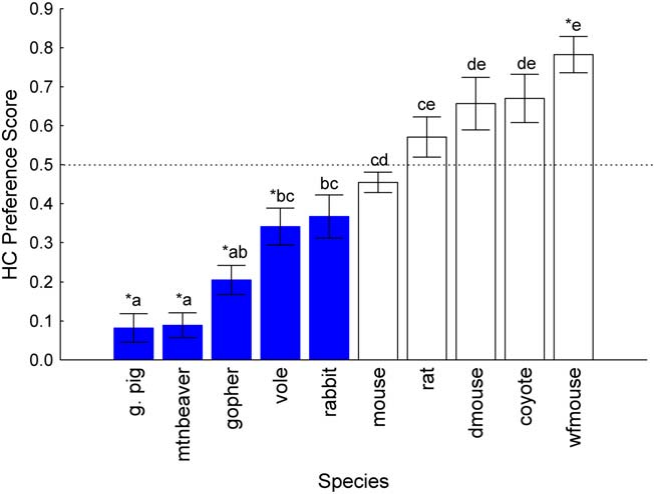
Hydrolyzed casein (HC) preference scores in two-choice tests with species organized in ascending order of magnitude. Scores represent g HC diet consumed/g total (HC+Cel) consumed; 4 d mean±SE; asterisks indicate significant difference from indifference (0.5) using a Dunn-Sidak corrected alpha value criterion = 0.0051 for 10 comparisons; species that share a letter do not significantly differ from each other based on Tukey's HSD posthoc tests; blue solid bars are herbivores, open bars are omnivores.

Variation in HC preference scores could be attributed to species identity (*F_9,142_* = 24.98, *P*<0.001; Fig. 1), but not to sex (*F_1,142_* = 0.37, *P* = 0.546). There was no significant interaction between these two factors (*F_9,142_* = 0.48, *P* = 0.889). Guinea pigs, mountain beavers, gophers and voles consumed less of the HC relative to the control (operationally defined as “avoidance”; all *t_15_* stats <−3.35, *P*s<0.0051), white-footed mice consumed more of the HC relative to the Cel (operationally defined as “preference”; *t_15_* = 6.06, *P*<0.0051), and the remaining species did not show statistically significant differences between the two alternatives (*P*>0.0051, Fig. 1).

**Figure 2:**
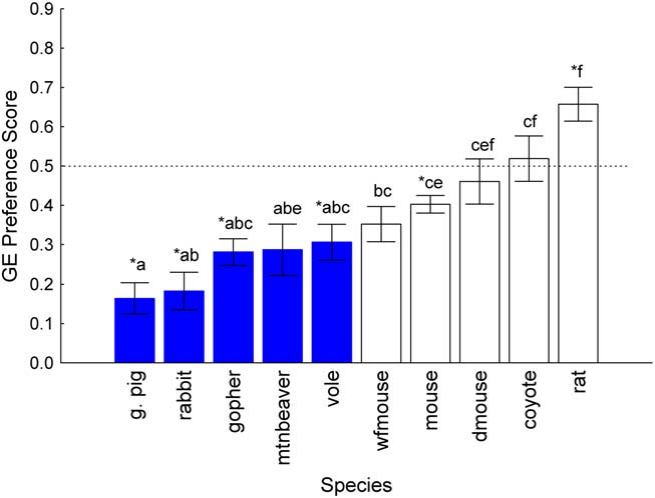
Gelatin (GE) preference scores in two-choice tests with species organized in ascending order of magnitude. Scores represent g GE diet consumed/g total (GE+Cel) consumed; 4 d mean±SE; asterisks indicate significant difference from indifference (0.5) using a Dunn-Sidak corrected alpha value criterion = 0.0051 for 10 comparisons; species that share a letter do not significantly differ from each other based on Tukey's HSD posthoc tests; blue solid bars are herbivores, open bars are omnivores.

Species also differed in their responses to GE and its control (*F_9,138_* = 10.40, *P*<0.001, Fig. 2). Sex (*F_1,138_* = 0.03, *P* = 0.868) and the interaction between species and sex (*F_9,138_* = 1.01, *P* = 0.439) could not explain variance in GE scores. Guinea pigs, rabbits, gophers, voles, and house mice avoided the GE (*t*s<−4.07, df: 14–17, *P*s<0.0051), while rats preferred it (*t*_15_ = 3.58, *P* = 0.0027). Mountain beaver, white-footed mouse, deer mouse and coyote GE preference scores were not statistically distinguishable from the null hypothesis of indifference (*P*s>0.0051, Fig. 2).

### Dietary niche and hydrolysate preference scores (4-d means)

**Figure 3:**
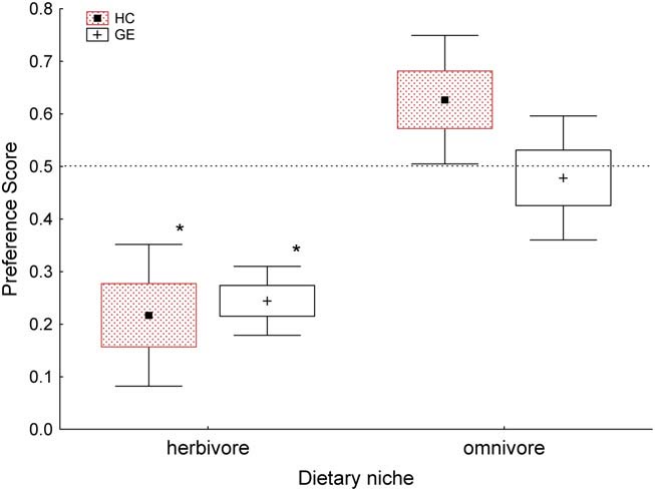
Herbivores, but not omnivores, avoid HC and GE. Herbivores (g. pig, mtnbeaver, gopher, vole, rabbit) differed significantly from omnivores (dmouse, coyote, mouse, rat, wfmouse) in scores for both hydrolysates (*P*s = 0.001); asterisks indicate significant (*P*s<0.015) differences from 0.5 (which would indicate no discrimination between the hydrolysate and the cellulose diet); boxes = means±SE, whiskers = means±SD.

The herbivores had lower preference scores for both hydrolysates than the omnivores (*t*_8_ = −5.0, *P* = 0.001 for both HC and GE t-tests, since rank distributions were the same for both dietary groups for both hydrolysates, Fig. 3). Herbivores avoided both hydrolysates (HC: *t*_4_ = −4.15, *P* = 0.014; GE: *t*_4_ = −7.74, *P* = 0.001), while omnivores showed neither avoidance nor preference for either hydrolysate (HC: *t*_4_ = 2.30, *P* = 0.083; GE: *t*_4_ = −0.40, *P* = 0.709).

### Daily preference scores for HC and GE

**Figure 4:**
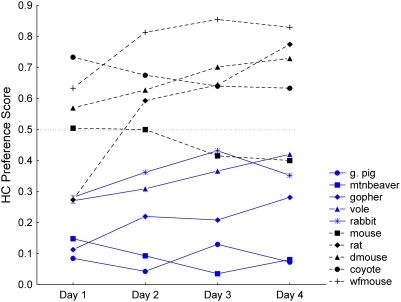
Daily HC preference scores. Species means for each day of testing. Blue symbols (solid lines) are herbivores; black symbols (dashed lines) are omnivores.

**Figure 5:**
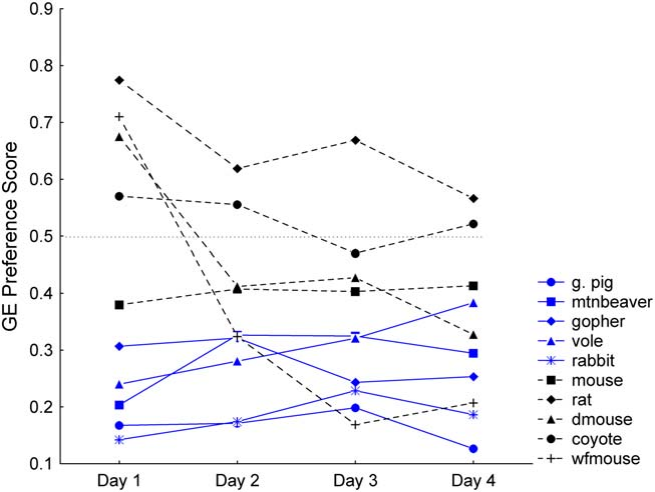
Daily GE preference scores. Species means for each day of testing. Blue symbols (solid lines) are herbivores; black symbols (dashed lines) are omnivores.

When daily HC and GE preference patterns were examined, most, but not all, of the species showed consistency in their scores across the four days of testing (Fig. 4 and Fig. 5). The differences between days 1 and 2 would be the most appropriate comparison for capturing the period during which the transition likely occurred between when sensory characteristics would have been the primary qualities of the diets that affected ingestion and when association of post-ingestive feedback to sensory characteristics might alter initial preferences. When days 1 and 2 were compared for the HC scores, there was an effect of species (*F_9,150_* = 19.18, *P*<0.001), day (*F_1,150_* = 9.15, *P* = 0.003) and an interaction between species and day (*F_9,150_* = 3.76, *P*<0.001). Day 2 HC preference scores (0.43, SE 0.03) were generally higher than on the first day (0.36, SE 0.03; Fig. 4), although only for the rat was there a significantly higher day 2 score (Tukey HSD, *P*<0.001; remaining species×interaction post-hoc tests showed no significant difference between days).

For the comparison of the GE scores on the first two days, species (*F_9,145_* = 12.97, *P*<0.001), day (*F_1,145_* = 9.38, *P* = 0.003) and an interaction between species and day (*F_9,145_* = 6.75, *P*<0.001) were statistically significant. For this hydrolysate, the mean day 1 scores were higher than day 2 scores (0.42, SE 0.03 versus 0.35, SE 0.02). Specifically, the deer and white-footed mice decreased their GE preference scores between day 1 and 2 (Tukey HSD tests; dmouse: *P* = 0.011, wfmouse: *P*<0.001), while changes in scores between the two days were not statistically significant in the other species (Fig. 5).

### HC versus intact casein (C) preference tests for three mouse species

Because protein requirements may have exaggerated mouse preferences for the hydrolysates when Cel was used as the control with a 24-h test paradigm, the three mouse species were tested with HC and intact casein (C). C was presented as the alternative option to HC in order to provide a control having very similar nutritional quality yet different sensory qualities from HC. When C was used as the control, overall mean 4-d HC preference scores were lower than for the 4-d means from the HC v Cel series (*F_1,47_* = 9.32, *P* = 0.004). The effect, however, depended on the species of mouse (test×species: *F_2,47_* = 3.23, *P* = 0.048). In posthoc tests, only the white-footed mice showed a statistically significant drop in their HC scores in the HC v C test compared to the original test. In general, species identity could explain variation in HC responses (*F_2,47_* = 13.06, *P*<0.001), which was due to the *Mus* having lower scores than the two *Peromyscus* (Tukey HSD: pairwise *P*s<0.001). The house mice were indifferent to the HC when it was paired with Cel (Fig. 1), but consumed significantly less HC than the control when C was used (*t*_17_ = −4.89, *P*<0.001). Deer mice consumed similar amounts of HC as the control in both testing series (Fig. 1; HC v C: *t*_15_ = 1.63, *P*>0.05). For the white-footed mouse, HC was preferred over the 4 d that it was paired with Cel (Fig. 1), but no longer so during the 4-d HC v C series (*t*_15_ = 0.40, *P*>0.05).

**Figure 6:**
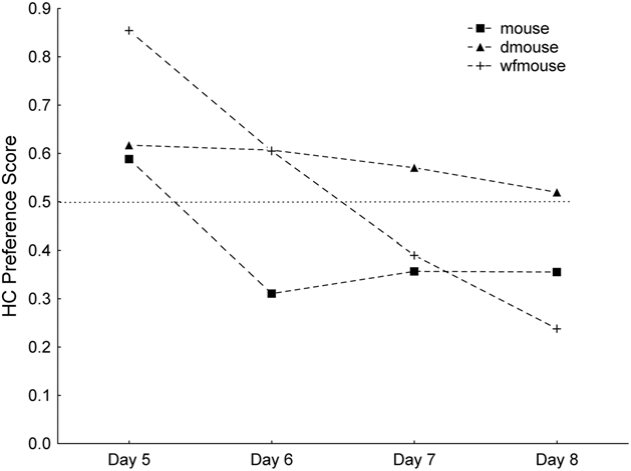
Mouse HC preference scores in follow-up HC v intact casein (C) tests. The dotted line at 0.5 represents indifference between the choices. The four days are labeled Day 5–Day 8 to avoid confusion with the HC v Cel series (Fig. 3).

When the daily preference scores were examined for the HC v C series, the deer mice maintained consistent HC scores across days, while the other two species decreased their scores after the initial day, which the wfmouse continued to do throughout the series (Fig. 6). Examination of HC scores for the first two days (“day 5” and “day 6,” Fig. 6) revealed statistically significant differences among species (*F_2,44_* = 9.84, *P*<0.001), between days (*F_1,44_* = 31.03, *P*<0.001) and a species×day interaction (*F_2,44_* = 3.98, *P* = 0.026). Both the house mice and white-footed mice had lower scores on day 6 than on day 5 (Tukey HSDs, *P*s<0.001), while deer mice maintained similar scores on both days (*P*>0.05).

### Exposure of HC or C on subsequent HC versus C preference tests in guinea pigs

**Figure 7:**
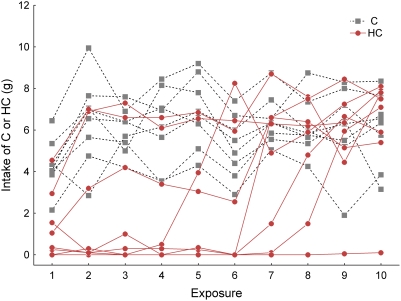
Individual guinea pig intake of either HC or C diets during exposure sessions. Rose circles and continuous lines depict individuals exposed to HC during a 4–5.5 h period in which the HC diet was the only food available; C-exposed animals are depicted with grey squares and dashed lines.

**Figure 8:**
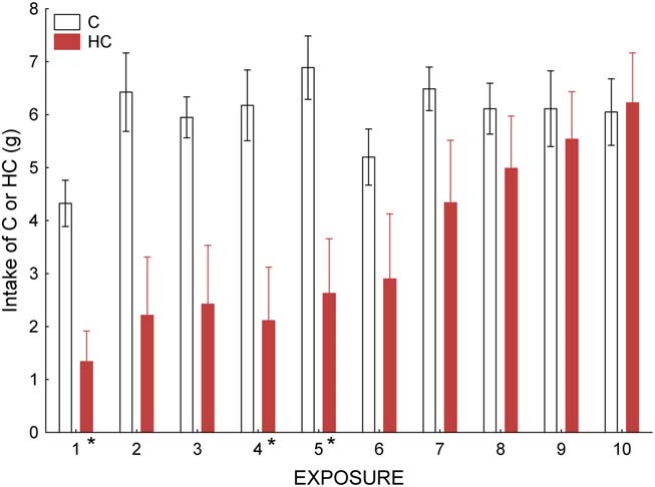
Mean (SE) guinea pig intake of either HC or C diets during exposure sessions. Solid rose bars show HC-exposed animals (N = 8) and open bars show C-exposed animals (N = 8). Asterisks indicate days for which there were significant statistical differences, correcting for multiple comparisons, between the HC- and C-exposed means.

**Figure 9:**
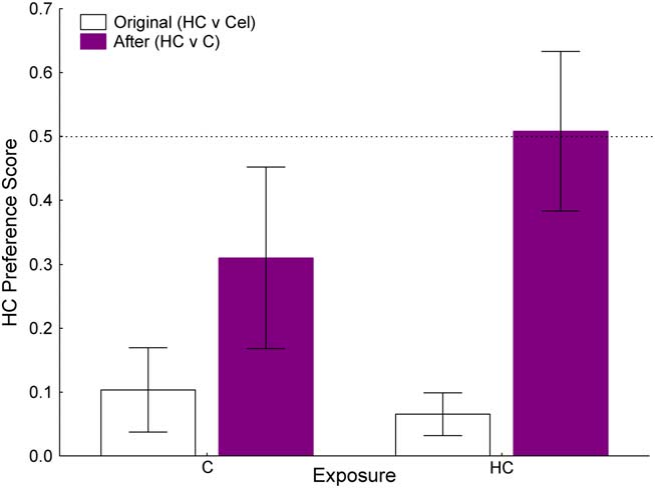
Mean (SE) guinea pig preference scores for HC v Cel (open bars) and for HC v C (purple bars). Individuals (N = 8) that were exposed to C between the two preference tests are shown on the left side of the figure; individuals (N = 8) that were exposed to HC between the two preference tests are shown on the right side of the figure. Regardless of exposure type, HC preference scores increased following exposure sessions (see text for details).

The second HC v C follow-up experiment was motivated by the observation that some species may have particularly long periods during which they sample novel foods before they actually incorporate the food into their diets [35]. The HC diet, which is characterized, at least by humans, by strong olfactory and gustatory properties may have been avoided by some of the herbivores because of food sampling strategies that may function to diminish negative effects of toxins found in plants [36]. We examined the effects of repeated exposures to HC on the guinea pigs, the species showing the strongest HC avoidance. The same individuals that had been tested earlier were allowed 10 exposures of mean duration of 4.7 h over a 22 d period to either the HC diet (N = 8) or a C diet (N = 8). Results showed that there was considerable individual variation in intake of the two diets (Fig. 7). In general, the C diet was readily consumed by the second exposure, while some of the HC-exposed animals needed four or more exposures to consume similar amounts as the C-exposed animals. One individual refused to consume the HC diet throughout the 10 exposure sessions, preferring not to eat at all. On average, there was a significant difference between the groups (*F_1,14_* = 7.30, *P* = 0.017), a significant effect of exposure number (Pillai's *F_9,6_* = 6.31, *P* = 0.018), and a significant interaction between these two factors (Pillai's *F_9,6_* = 6.89, *P* = 0.015). When the intake of HC and C were compared for each exposure session, intake was not significantly different after the fifth exposure (*P*s>0.107). For the first five exposures, the differences between the mean HC and C intake on day 1, 4 and 5 were statistically significant (*T*_14_s>3.36, *P*s<0.005), but the differences on days 2 and 3 did not meet the adjusted alpha criterion (k = 10 comparisons, α′ = 0.0051; *P*_d2_ = 0.007, *P*_d3_ = 0.009; Fig. 8). Following the exposures to either the HC or the C diets, the guinea pigs' HC preferences were again examined, but in this case the two-choice test paired the HC and C diets (Fig. 9, solid bars). When these were compared to the animal's original HC preference scores (from the HC v Cel tests; Fig. 9, open bars), there was a difference in the 4-d mean scores with the HC v C series showing increased HC preference scores (*F_1,14_* = 16.72, *P* = 0.001). There was no significant effect of exposure diet (HC or C; *F_1,14_* = 0.77, *P* = 0.395) and no diet×test (original or HC v C) interaction (*F_1,14_* = 2.12, *P* = 0.168).

## Discussion

### Taxonomic variability in response to protein hydrolysates

Greater consumption of the hydrolysate diets, relative to the cellulose diet, was uncommon among the species tested in this study. Of the 10 species, only the white-footed mouse preferred the HC over the Cel diet, and only the rat preferred the GE over the Cel diet. The remaining species were indifferent to or avoided the hydrolysates. Further, the range of responses could be categorized by the species' basic dietary niches: in general, omnivores were generally indiscriminate toward food containing hydrolyzed protein while herbivores avoided it.

Why herbivores, but not omnivores, would avoid hydrolysates is unknown. Hydrolyzed protein, as mixtures of peptides and amino acids, are typically encountered on degrading substrates (e.g., animal or plant tissue injury and carcasses). The sensory cues emitted from them may signal a “non-food” or a “danger” for ingestion that could be detrimental to an herbivore's fitness. However for omnivores, even if the proportion of diet items that may be associated with protein hydroysis is small [37 and sources listed in Supporting Information Appendix S1], these items could still confer selective advantages to opportunistic omnivores that exploit them. Obligate carnivores consumed over 10 times the amount of a 3% (the highest concentration tested) HC than the water diluent control [17], which is consistent with the hypothesis that ingestive response to protein hydrolysates is related to the typical proportion of animal products in the diet. Additional data on felines and on additional herbivorous and omnivorous species, which ideally would control for taxonomic relatedness, would be needed to evaluate this hypothesis.

### Potential mechanisms of hydrolysate avoidance

This study found that most species' preference scores were relatively consistent for each of the four days of the HC and GE series. In general, HC preference scores increased on the second day of testing while GE scores dropped on day 2. Among the species in our study, rats had the strongest increase in HC preference over the two days and the two *Peromyscus* mice (deer mouse and white-footed mice) had largest decrease in GE preference.

These data from the first two days of testing are consistent with the hypothesis that nutritive feedback was positive for HC and negative for GE. HC, originating from milk, is well-known for being a high-quality nitrogen source, while GE, originating from collagen, is completely deficient in tryptophan and provides only small amounts of other essential amino acids [28]. Ability to detect and respond to essential amino acid composition in foods is found in many species, and rats, as a mammalian model, have avoided deficient foods within 30 minutes under a variety of scenarios [reviewed in 38], which could be mediated by the formation of a conditioned aversion to the deficient diet and/or changes in meal patterns [39], [40]. Studies by other investigators have found that GE, when compared to casein controls, induced weight loss and food intake reduction in rats, which was associated with increased central nervous system histamine receptor (H_1_) levels [29] and lower plasma concentrations of growth hormone and essential amino acids [30].

Our rats did not show a statistically significant drop in their day 2 GE scores and were the only species to show, averaged over the 4 d, a preference for GE. The rats' intake was not depressed during the GE tests (mean intake during GE tests: 8.2 g/test, SE 0.4; intake during HC tests: 7.7 g/test, SE 0.77). We also found no evidence of the rats (or any of the other lab animals, for which we monitored body weights) being stressed by the GE (or any of the other experimental procedures) in a way such that they were unable to maintain their body weight, and in fact most individuals among the laboratory species tested gained weight through the study (Supporting Information, Fig. S1, S2). One possible reason for why rats preferred GE in our experiments is that GE, although being a poor protein source, would have been better than the Cel alternative. Another possibility is that the access to the nutritionally complete chow, following the 4-h test, may have protected the rats from deficiency-related negative feedback.

For mice, our follow-up experiment with HC suggests that sensory qualities do play a large role in feeding decisions for two of the species. When the nutritional value of an HC diet and its alternative (C diet) were comparable, we observed avoidance of HC by the end of the HC v C series by the white-footed and house mice. In the HC v Cel series, the white-footed mice had preferred HC and the house mice had been indifferent to the HC. Deer mice, however, do not appear to be deterred by the sensory properties of HC. Perhaps once deer mice learn that a food is safe and nutritious, particularly if the learning takes place in a context in which this food is the best nutritive option, they are slow to drop this food from the diet, even when another food, with different sensory qualities, offers a nutritionally equivalent reward. Flavor preferences that have been learned by positive association with nutrients can persist for long periods after the nutritive reward is no longer coupled to the cue [e.g., 41].

The guinea pig HC follow-up experiment further suggests that ingestive experience with HC can increase its consumption in later feeding bouts. Food neophobia [42] can inhibit ingestion of new foods, although the time frame for regular incorporation into the diet may take much longer (days or weeks) for some species than has typically been documented for “neophobia” [35]. Guinea pigs that were encouraged, by not providing other food sources, to sample the HC diet in multiple sessions prior to a preference test between the HC diet and an intact C diet consumed similar amounts of HC and C in the preference test. Thus, it appears that repeated exposure to the HC, available as a single choice, reduces avoidance behavior in later choice situations, presumably because the animals learn to associate positive nutritive value with the strong, initially aversive, chemosensory cues. Conservative food sampling strategies based on chemosensory cues may have evolved in herbivores to reduce the risk of over-ingesting toxic defense chemicals commonly found in plants [36].

Further, C was completely novel to the animals, while the HC diet had been previously experienced in the HC v Cel series. The extra time necessary for the guinea pigs exposed to the HC diet to match the intake of the guinea pigs given the C diet (Fig. 8) suggests that HC's sensory profile is less appealing to guinea pigs than intact casein. A preference for C over HC is consistent with direct and indirect evidence from some other species (humans [10], [43], mice [16], rats [15], deer [12]).

Notably, even those individuals that had been exposed to the C diet showed greater willingness to eat the HC diet during the two-choice tests. C appears to have some sensory characteristics that guinea pigs generalize to the HC. This hypothesis would have to be tested experimentally, by for example including a group exposed to another alternative, since it is possible that ingestive experience with any food with different sensory profiles than their typical chow would stimulate more experimentation in later choice situations [44]. Interestingly, repeated HC dietary experience may in itself also broaden later dietary breadth, at least in humans [45].

Ingestive experience with C and HC may not have altered the original preference of Cel over HC exhibited by these guinea pigs. The Cel diet could have been preferred originally, not only because of aversive properties of HC, but also because of attractive properties of Cel. For example, some herbivores may be able to gain some nutritional value from cellulose [23]–[27], and/or the Cel diet may have more closely resembled plants and plant-based foods on various nutritional, sensory and familiarity dimensions to these animals. Also, the fasting component of the testing may have altered preferences for macronutrients during recovery [46], [47].

### Implications for laboratory models and the protection of agricultural resources

The white-footed mouse appears to be very responsive to HC's nutritive value. It had the highest preference scores of any of the species when HC was paired with the nutritionally poor Cel, yet it strongly avoided HC once another equivalent protein-source, C, became available. This suggests that this species is also quite sensitive to HC's deterrent sensory properties. Of the species we tested, the white-footed mouse would be the best for investigating sensory mechanisms of HC rejection. If the white-footed mouse were to also avoid some of the bitter peptides in HC that humans find distasteful [e.g., 5], [10], [48], it may be useful in the ongoing work on designing better tasting HC products. Guinea pigs showed strong avoidance of both hydrolysates, which for the HC at least, they overcame with ingestive experience. Given that most of the herbivores also avoided the GE, and GE avoidance has been attributed to negative post-ingestive feedback in some previous studies, combining HC and GE may be more effective than HC alone in protecting agricultural resources. Specific experiments would need to be conducted to determine the conditions under which hydrolysates would be most effective as repellents [e.g., 14].

## Materials and Methods

### Animals and housing

Individuals from 10 species (N = 16–18 per species), representing three mammalian orders (Table 1), were tested in a series of two-choice preference tests to assess their willingness to consume two protein hydrolysates (HC, GE). These species reflected a range of feeding ecologies from strict herbivory to scavenging/carnivory, and could be characterized as representing nuisance species relevant to the management of human-wildlife conflicts (mountain beaver, pocket gopher, vole, rabbit, coyote) and/or standard biomedical laboratory model species (mice, rats, guinea pigs). Depending on the species, animals were wild-caught, purchased or part of an existing captive colony (Table 2).

**Table 2:** Sample sizes by sex, sources and test sites for species used in preference tests.

Short name	Female	male	Source (strain if applicable)	Test Site
mtnbeaver	6	10	wild caught	Olympia, WA^3^
coyote	5	11	captive colony	Logan, UT^3^
g. pig	8	8	purchased^1^ (Hartley)	Philadelphia, PA^4^
vole	8	8	wild caught	Olympia, WA
mouse	9	9	purchased^1^ (CD-1)	Philadelphia, PA
rabbit	8^a^	8	purchased^1^ (New Zealand White)	Ft. Collins, CO^3^
wfmouse	8	8	purchased^2^	Philadelphia, PA
dmouse	8	8	purchased^2^	Philadelphia, PA
rat	8	8	purchased^1^ (Long-Evans)	Philadelphia, PA
gopher	8^b^	8^b^	wild caught	Olympia, WA

a N = 7 females for GE tests.

b N = 9 females, N = 7 males for GE tests.

1 Charles River Laboratories, Wilmington, MA.

2 Peromyscus Genetic Stock Center, Univ. SC, Columbia, SC.

3 USDA/APHIS/WS/NWRC facility.

4 Monell Chemical Senses Center.

All animals were housed individually at the facility at which they were tested (Table 2). Three species were caged outdoors under large roofs, such that the cages received some protection from sun and precipitation but had no access to natural forage, (enclosure sizes: rabbit: 2.4 m×2.4 m×3 m, mountain beaver: 2.4 m×2.4 m×1.2 m, coyote: 1.2 m×3.7 m×1.8 m or 1.2 m×2.4 m 1.8 m). The remaining species were housed within indoor animal facilities in plastic tubs fitted with stainless steel cage tops (pocket gopher: 27.9 cm×35.6 cm×17.8 cm; vole: 20.3 cm×30.5 cm×12.7 cm; rat: 30 cm×35 cm×15 cm; three mouse species: 17 cm×26.5 cm×12 cm) or in a stacked, stainless steel caging unit (guinea pig: individual units 49 cm×55 cm×29 cm). Five of these indoor species were maintained on a 12L∶12D cycle, with lights on at 0815 h for the guinea pigs and rats and at 0700 h for the three mouse species. The fossorial gophers were housed in the dark, except for when lighting was required for staff to perform feedings (<20 min/day) and once weekly cage changes (∼1 h). Voles were kept in a room with some natural lightening through windows, which was supplemented by artificial lighting between 0730 h and 1600 h. Throughout the duration of the study, animals had ad libitum access to water.

The laboratory species housed at Monell were weighed regularly during the study (mice: every 2–3 days; rats and guinea pigs: once weekly). All procedures were approved by the Monell Chemical Senses Center Institutional Animal Care and Use Committee (ACC #1115) or the National Wildlife Research Center Institutional Animal Care and Use Committee (QA-1372, QA-1461).

### Diets

There were four categories of diets: maintenance, training, test and follow-up diets. Maintenance diets were commercially purchased complete diets (Table 3), which were available during the study when animals were not fasting or undergoing preference tests. Mountain beavers, pocket gophers, voles, and rabbits were supplemented with an apple each day, and mountain beaver and pocket gopher received alfalfa cubes in addition to the apple, as was standard procedure in U.S. Department of Agricultural facilities to ease the stress for wild animals held captive. Training diets (compositions, which differed among species, are listed in Table 4) were used to give the animals experience with the feeding schedule and feeding containers prior to the testing with the test diets.

**Table 3:** Maintenance diets provided ad libitum to subjects.

Species	Maintenance Diet	Basic Composition^6^
deer mouse	Rodent Diet 8604^1^	CP = 24%, F = 4%, Fib = 4.5%
gopher	Rat Diet 5012^2^	CP = 22%, F = 4%, Fib = 5%
house mouse	Rodent Diet 8604	CP = 24%, F = 4%, Fib = 4.5%
mountain beaver	Rat Diet 5012	CP = 22%, F = 4%, Fib = 5%
rabbit	Rabbit Chow Complete Plus^3^	CP = 16%, F = 1.5%, Fib = 24%
rat	Rodent Diet 8604	CP = 24%, F = 4%, Fib = 4.5%
vole	Rat Diet 5012	CP = 22%, F = 4%, Fib = 5%
white-footed mouse	Rodent Diet 8604	CP = 24%, F = 4%, Fib = 4.5%
guinea pig	Guinea Pig Chow 5025^4^	CP = 18%, F = 4%, Fib = 16%
coyote	Carnivore Diet^5^	CP = 37%, F = 18%, Fib = n/a

1 Harlan Teklad (Madison, WI).

2 LabDiet (PMI Nutrition International, Richmond, IN).

3 Purina Mills (LLC, St. Louis, MO).

4 Dyets, Inc (Bethlehem PA).

5 Fur Breeders Agriculture Cooperative (Sandy, UT).

6 Manufacture's guaranteed analysis (CP = minimum crude protein; F = minimum crude fat; Fib = maximum crude fiber).

**Table 4:** Composition of training and test diets (g/kg; A, B, or C diets used depending on species).

Ingredient	(A)Training	(A)Test	(B)Training	(B)Test	(C)Test
Sucrose^1^ ^ or ^ 4	300	300	356	356	300
Starch^1^	250	250	100	100	
Flour^3^	350	150			
Oil^1^ ^ or ^ 2	55	55	100	100	
AIN salt mix^1^	35	35			
AIN vitamin mix^1^	10	10			
Cellulose^1^			100	100	
Guar gum^1^			50	50	
Salt mix^1^			75	75	
Vitamin mix^1^			10	10	
Ascorbic acid^1^			4	4	
Methionine^1^			3	3	
Choline^1^			2	2	
Hydrogenated oil^1^					500
Soy protein^1^			200		
Test substance (1 per test diet)					
HC^5^		200 or		200 or	200 or
GE^6^		200 or		200 or	200 or
Cellulose^1^		200		200	200

Species given (A) diets: mountain beaver, vole, house mouse, rabbit, white-footed mouse, deer mouse, rat, gopher.

Species given (B) diets: guinea pig.

Species given (C) diet: coyote.

1 Dyets, Inc (Bethlehem PA); also mixed Series B diets using author-provided HC^5^ & GE^6^.

2 Crisco (The J.M. Smucker Co., Orrville, OH).

3 King Arthur, whole wheat flour (Norwich, Vermont).

4 generic table sugar (Kroger or Pathmark grocery store labels).

5 HCA-411 hydrolyzed casein, American Casein Company (Burlington, NJ).

6 PolyPro5000 gelatin, PB Leiner (Davenport, IA).

Test diets included an HC-diet (20% w/w), a GE-diet (20% w/w), and a cellulose diet (Cel) in which the 20% hydrolysate part of the diet was replaced with cellulose. The ingredients comprising the remaining 80% of these test diets were identical for each type of test diet for a particular species. However the composition of this 80% base mixture differed among some of the species in order to accommodate differences in feeding behavior and the particular setups of feeding containers in their cages (Table 4). In other words, although test diets among species sometimes differed, they always contained the two hydrolysates of interest (HC, GE) and the Cel alternative, which were the only ingredients that then differed among a particular species' three test diets. References to a particular test diet will identify it by its distinguishing ingredient (HC, GE, or Cel).

For diets that were not commercially purchased (Table 4, Series A), ingredients were added to a mixer (Professional 5 Plus, KitchenAid, USA, St. Joseph, MI or Hobart N-50 and I-300 mixers, Hobart manufacturing Co., Troy, OH) with enough water to produce a dough (water volume varied with local facility conditions: 133–145 ml per kg for HC, 100–130 ml per kg for GE, 410–550 ml per kg for Cel, and 200–260 ml per kg for the training diet). The dough was rolled out into 1–2 cm slabs, cut into 8 cm×4 cm or smaller pieces, and dehydrated at approximately 66.5 C (D10 food dehydrator, The SausageMaker, Inc., Buffalo, NY) until a constant mass was reached. Dried pieces were further cut as necessary to make “pellets” of as uniform size as possible that were the appropriate size for the species to be tested (e.g., approximately 1–2 cm cubes for mice). The coyote test diet ingredients (Table 4, Series C) were mixed until blended and stored in plastic tubs at room temperature until they were needed.

Test diets were presented in bowls placed on the cage bottom for rabbits and mountain beaver. The pocket voles, gophers, rats and mice species' test diets were placed in the wire cage top feeding troughs that suspended the foods into the cages, and which had been divided in half for simultaneous access to two test choices. Guinea pigs received their test diets in two J-shaped feeders that had been attached to the back of the cage (Gravity Bin Feeder, Super Pet, Pets Int'l, Elk Grove Village, IL). Test diets for the coyotes were tamped into 15.3 cm long×5.1 cm (outside diameter) polypropylene pipes (schedule 80), which were open at both ends for access to the mixtures. Follow-up diets are described below with the follow-up test procedures.

### Hydrolysate preference tests using all species (HC v Cel, GE v Cel)

All feeding procedures were conducted in the animals' home cages or enclosures between May 29, 2006 and April 6, 2007 at one of the four facilities involved in the testing (Table 2). For each species, diet manipulations occurred over three weeks. In the first week, training diets were used to familiarize the animals with the feeding containers and schedule. Animals received test diets during week 2 (HC v. Cel) and week 3 (GE v Cel).

To accommodate differences in species biology, we used three different schedules for collecting the data. Most species (vole, gopher, mountain beaver, rabbit, rat, guinea pig) were tested using the first schedule. For this schedule, maintenance diets were removed on Monday evening to initiate an overnight 14-h fast to motivate consumption during the following day's preference tests. On Tuesday morning, animals were given four hours of access to the test diets (either HC v Cel or GE v Cel; hereafter called “preference tests”), which was then followed by six hours of access to maintenance diet. Tuesday evening, the maintenance diet was removed to initiate the 14-h fast preceding Wednesday's 4-h preference test, which was again followed by 6 h of maintenance diet access. This cycle continued through Friday afternoon's maintenance diets, to which animals continued to have access throughout the weekend, until the maintenance diets were again removed Monday evening. Thus, for each hydrolysate, there were four preference test sessions of 4 h duration each.

A second schedule was used for the three mice species. Because of the possibility that the physiological consequences of fasting would be comparatively severe for these small rodents with high metabolic rates (e.g., torpor-like cardiovascular responses within 6 h fasting, [49]) we provided the mice ad libitum access to the test diets. Thus, the mice species also had four preference tests per hydrolysate, but each was 24 h in duration. Further, the mice only received maintenance diet between the end of preference tests on Friday and the beginning of the following week's preference tests on Monday.

The final experimental schedule was used for the coyotes. Coyotes have a tendency to eat large amounts of food quickly, which may also limit the exposure to sensory stimuli they receive if food is not retained in the mouth for long [50]. For this reason, coyotes were tested after they had received their morning meal (1.25–2.25 h following feeding of maintenance diet) using test diets and feeding tubes that required some handling time (food could be licked out or displaced with some oral or paw manipulation of the plastic tube). The training week was used to familiarize the coyotes with the feeding tubes, as well as to determine the optimal duration for their preference tests. To do this, the coyotes were given a single tube filled with maintenance diet for 4 d of the training week (D1: 90 min, D2: 60 min, D3–4: 15 min). Maintenance diet was used instead of a training diet because we were trying to determine a test duration that was short enough that the animals would not be able to deplete the tubes, even when filled with a familiar, palatable diet. Preference test durations of 15 min allowed the animals time to interact with the feeding tubes, but typically not enough time to empty them.

We tested each hydrolysate-cellulose pair over 5 d (Monday–Friday), since some coyotes were easily distracted from the feeding tubes by external stimuli outside of our control (e.g., howling in other pens). However, only the first four days' data were analyzed in order to make the number of tests and experience with the hydrolysates more compatible with the other species. For the analyses that used four-day mean preferences for each hydrolysate series, the fifth day's data was used in three cases when the individuals did not interact with the stimuli on one of the earlier days and consequently were missing a preference score for a day. Since we were using coyotes that were part of a permanent captive colony, we did not alter their established feeding schedule of a single ration of maintenance diet per day for all days but Sundays.

For all species, the position of the feeding container (left or right) in which the hydrolysate was presented in the initial preference test was randomized for each individual and then alternated thereafter for the remaining sessions for that hydrolysate in order to counterbalance for any positional biases an individual may exhibit.

### HC versus C preference tests for house, white-footed and deer mice

These three species were given an additional two-choice preference series to assess the robustness of their results from the HC (v Cel) preference tests, in which the HC scores may have been elevated given that the HC diet was the only available source of protein over the 4 days of testing. We paired AIN-93G Purified Rodent Diet (Dyets Inc., Bethlehem, PA), of which casein comprises 20%, with an HC diet that was identical to the AIN-93G diet except that HC (HCA-411, American Casein Company, Burlington, NJ) replaced the casein (substitution performed by Dyets, Inc.). By providing these nutritionally similar diets that differed only in the degree of hydrolysis of the protein source, the mice would presumably base their selections primarily on the chemosensory properties of the diets. These HC v C pairings were conducted in the same manner as the previous tests such that four 24-h preference scores were generated for the same mice that had been tested originally. The *Mus* follow-up HC v C preference tests were performed one week after completing their GE v Cel preference tests, and both *Peromyscus* species were tested 3 weeks after the completion of their GE v Cel tests. Between the GE trials and the HC v C follow-up tests, mice had ad libitum access to maintenance diet (Table 3) and water.

### HC or C exposure followed by HC versus C preference tests for guinea pigs

Using the species that showed the strongest avoidance of HC, we examined the role of learning and experience on HC consumption. The guinea pigs that had been tested in the original HC and GE series were randomly divided into two groups, each composed of four males and four females. One group was assigned the HC diet used for the original HC v Cel testing (Table 4, Series B, Test), while the second group was assigned to the same diet except that intact casein (C) replaced the HC portion of the diet (substitution performed by Dyets, Inc.; Bethlehem PA). On Mondays, Wednesdays and Fridays, for a total of 10 times, guinea pigs received either HC or C in place of their maintenance diet (Table 3) for 4–5.5 h in the afternoon. They had access to ad libitum water during this period (“exposure”), but the HC (or C) diet was the only food available during the exposures. No fasting occurred during the exposures. Following the 10 exposures and then 48 h of ad libitum maintenance diet, the guinea pigs were re-acclimated to the 14-h overnight fast, 4-h choice test (using two containers of maintenance diet) and 6-h of a single container of maintenance diet for four days. The final two-choice test series followed immediately after the re-acclimation to the fasting/testing schedule, such that the HC v C diet pairing was offered for 4 d in the same manner as the HC v Cel tests had been conducted. The follow-up exposures occurred 38 d after the last GE v Cel test and the last HC v C test occurred on April 19, 2007.

### Analyses

Test stimuli were weighed before and after the preference tests and the weight changes were used as estimates of the subjects' intake during the tests. Intake responses were standardized among species by using proportional intake of the hydrolysates (referred to as “preference scores”) that were calculated by dividing the intake of the hydrolysate diet by the total test diet intake for that session (HC+Cel or GE+Cel).

Four-day mean preference scores were calculated for each individual and then were arcsin square root transformed, which improved the fit of the data to the normality and variance assumptions of analysis of variance (ANOVA) models. We examined the effects of species, sex, and their interactions in a two-way ANOVA, with a separate model for each hydrolysate. Tukey's honestly significant difference (HSD) tests were used for post-hoc, pair-wise comparisons among species. To determine whether species preference scores could be interpreted as a hydrolysate preference (score>0.5), or avoidance (score<0.5), the 4-d mean preference scores were compared to 0.5 using a one-sample t-test with the alpha-level criterion adjusted for multiple comparisons using the Dunn-Sidak procedure [51]. This correction for the 10 species comparisons to a null hypothesis of indifference resulted in an α′ = 0.0051 for the *p*-value to be considered significant. Temporal patterns in hydrolysate preference were examined by comparing preference scores on the first and second days of the HC and GE series using Repeated Measures (RM) ANOVA models with species as the between-subjects factor and day as the within-subjects factor.

To answer the question of whether ingestive responses to the hydrolysates may have been associated with the dietary niche of the species, we grouped the species into the category of herbivore or omnivore (N = 5/group) based on recorded diets in natural populations (Supporting Information Appendix S1). Within each dietary group, we ranked the species by their mean hydrolysate preference score and used these ranks to conduct a two-sample t-test assuming unequal variance [52]. To determine whether herbivores and omnivores avoided the hydrolysates, means for each dietary group were calculated using the mean 4-d preference scores of the five species in each group. These group means were interpreted as hydrolysate avoidance (<0.5) or preference (>0.5) based on the results of one-sample t-tests against a null hypothesis of 0.5.

For the HC v C tests with the three species of mice, mean 4-d HC preference scores from the HC v C were compared with mean 4-d HC scores from the HC v Cel series using an RM ANOVA with species as the between-subjects factor and test series as the within-subjects factor. Mean 4-d HC preference scores from the HC v C series were statistically compared to a score of 0.5 (equal consumption of HC and C) using 1-sample t-tests for each species. Temporal patterns in HC scores in the HC v C series were examined by comparing preference scores on the first and second days of the series using RM ANOVA with species as the between-subjects factor and day as the within-subjects factor.

For the guinea pig HC v C experiment, intake of exposure diets (g HC and C) was examined using a RM ANOVA with group (HC or C exposed) as the between-subjects factor and exposure number as the within-subjects factor. Violations of the sphericity assumption, if they occurred, were dealt with by using Pillai's corrected F-values. The interaction between group and exposure day was interpreted by comparing the HC and C intake for each group for each of the 10 exposures (two-sample t-tests, α′ = 0.0051 for 10 comparisons). Comparison of the two-choice HC v C series to the original HC v Cel series was done using an RM ANOVA with group (HC or C exposed) as the between-subjects factor and 4-d mean for the test (original and HC v C) as the within-subjects factor.

All of the ANOVAs and t-tests were conducted on the transformed preference scores using Statistica [53]. All figures depict untransformed data, means and standard errors, unless otherwise noted.

## Supporting Information

Figure S1 Individual body weights (g) of mice. Top: Mus, middle: Peromyscus maniculatus, bottom: P. leucopus; species abbreviations are shown on the y-axis label (note different scales). Diets animals had been fed prior to each BW measurement: BW3–4 = Training; BW6–7 = HC, BW9–10 = GE; all others = Maintenance; BWs taken 2–3 d apart.
(1.99 MB TIF)

Figure S2 Individual body weights (g) of rats (top) and guinea pigs (bottom). BW1 was taken as a baseline, following arrival to the facility; BW2 was taken after the Training diets, BW3 was taken after the HC tests, BW4 was taken after the GE tests; BWs taken once per week; note the different y-axis scales. For the rats, all males were heavier than females by the end of the experiment (BW3 and BW4 measurements correspond to ages 5.5 and 6.5 weeks, when sexual size dimorphism develops for this strain of rat).
(1.88 MB TIF)

Appendix S1 Species diets determined from stomach contents, feces, and direct and indirect observation of feeding in free-ranging, wild populations.
(0.04 MB DOC)
