# Supplementary figures and images for: Protein Hydrolysates Are Avoided by Herbivores but Not by Omnivores in Two-Choice Preference Tests
Source: PLoS One. 2009 Jan 5;4(1):e4126. doi: 10.1371/journal.pone.0004126 (PMC2606031; doi:10.1371/journal.pone.0004126)

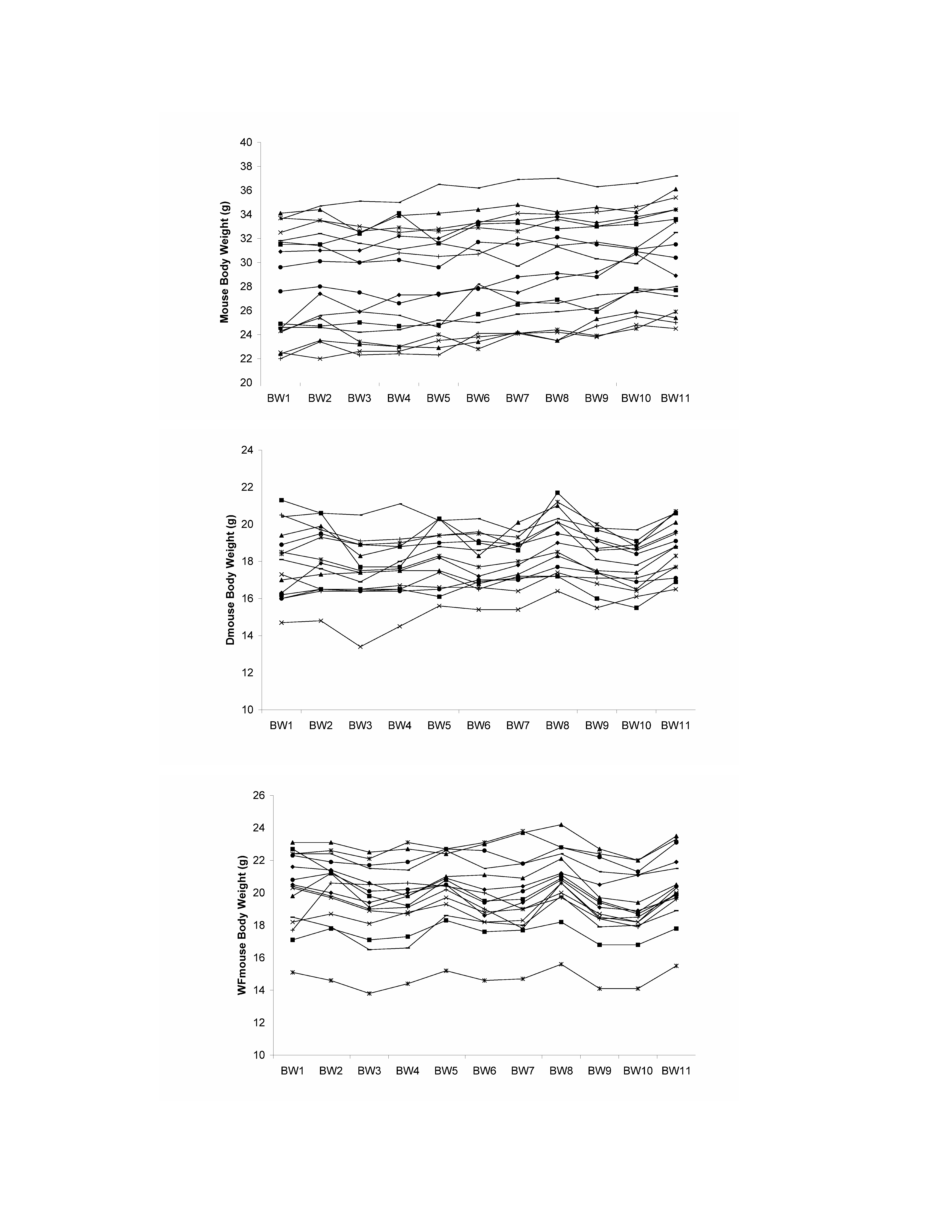

Supplement: Figure S1 — Individual body weights (g) of mice. Top: Mus, middle: Peromyscus maniculatus, bottom: P. leucopus; species abbreviations are shown on the y-axis label (note different scales). Diets animals had been fed prior to each BW measurement: BW3–4 = Training; BW6–7 = HC, BW9–10 = GE; all others = Maintenance; BWs taken 2–3 d apart. (1.99 MB TIF) [file pone.0004126.s001.tif]

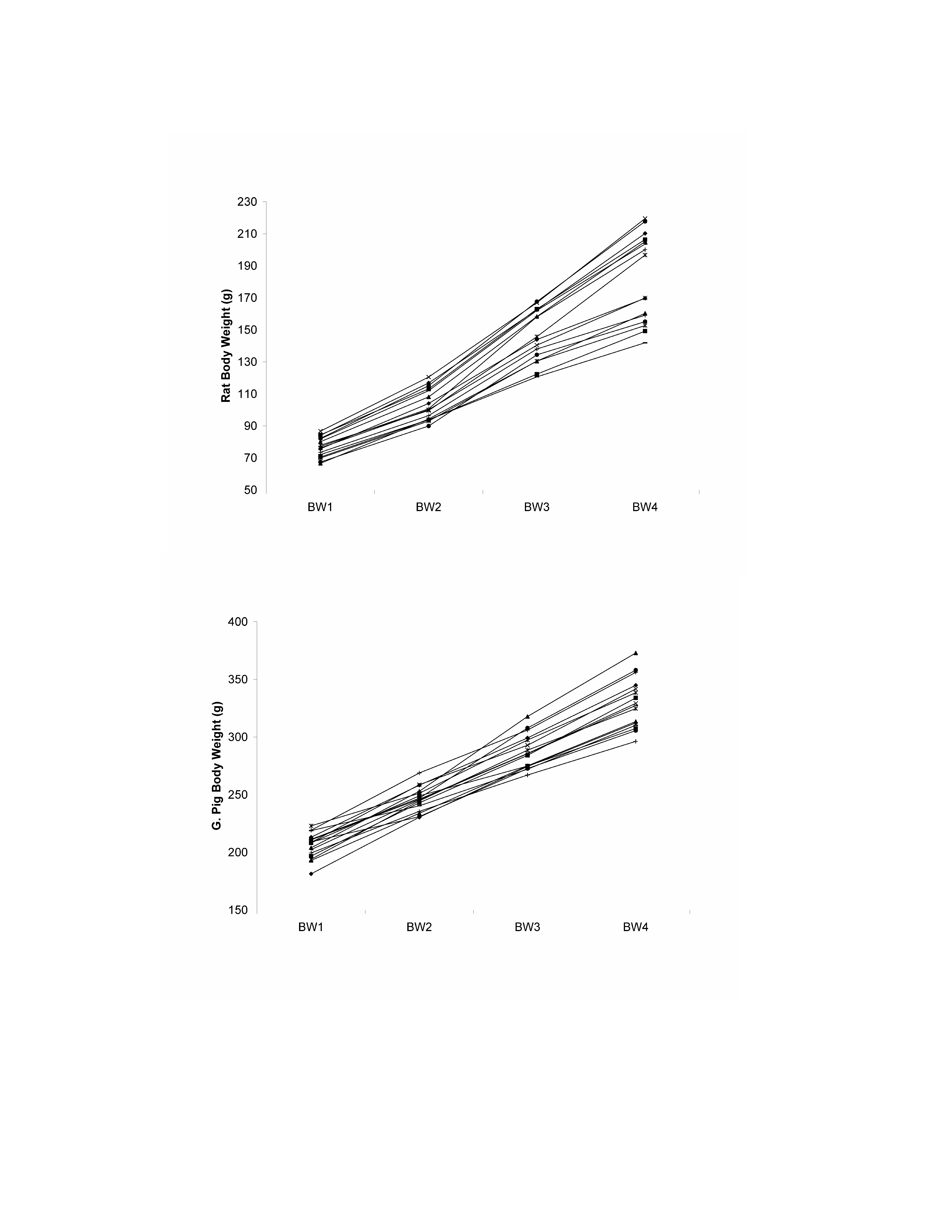

Supplement: Figure S2 — Individual body weights (g) of rats (top) and guinea pigs (bottom). BW1 was taken as a baseline, following arrival to the facility; BW2 was taken after the Training diets, BW3 was taken after the HC tests, BW4 was taken after the GE tests; BWs taken once per week; note the different y-axis scales. For the rats, all males were heavier than females by the end of the experiment (BW3 and BW4 measurements correspond to ages 5.5 and 6.5 weeks, when sexual size dimorphism develops for this strain of rat). (1.88 MB TIF) [file pone.0004126.s002.tif]
