# Supplementary material for: Protein Hydrolysates Are Avoided by Herbivores but Not by Omnivores in Two-Choice Preference Tests
Source: PLoS One. 2009 Jan 5;4(1):e4126. doi: 10.1371/journal.pone.0004126 (PMC2606031; doi:10.1371/journal.pone.0004126)
Supplement: Appendix S1 — Species diets determined from stomach contents, feces, and direct and indirect observation of feeding in free-ranging, wild populations. (0.04 MB DOC) [file pone.0004126.s003.doc]

**Appendix S1. Species diets determined from stomach contents, feces, and direct and indirect observation of feeding in free-ranging, wild populations.**

| Species | Group | Diet | Source |
| --- | --- | --- | --- |
| *Aplodontia rufa*  (mtnbeaver) | herbivore | ferns, forbs, shrubs, grasses, woody plants | [1] |
| *Canis latrans*  (coyote) | omnivore | fruit, plants, sunflower seeds, insects, mammals, birds | [2-4] |
| *Cavia porcellus*  (g. pig) | herbivore | grasses, dicotyledonous plants  (*C. porcellus* is a domesticated species; diets taken from wild relatives, *C. aperea*.) | [5,6] |
| *Microtus townsendii*  (vole) | herbivore | grassland and marsh vegetation, roots, woody plants | [7] |
| *Mus musculus*  (mouse) | omnivore | seeds, corn, vegetation (roots, stems, green leaves), invertebrates | [8] |
| *Oryctolagus cuniculus*  (rabbit) | herbivore | grasses, dicotyledonous plants | [9] |
| *Peromyscus leucopus*  (wfmouse) | omnivore | seeds and nuts, vegetation (roots, stems, green leaves), invertebrates | [8] |
| *Peromyscus maniculatus*  (dmouse) | omnivore | seeds, crops (soybean, corn), vegetation (roots, stems, green leaves), invertebrates, | [8] |
| *Rattus norvegicus*  (rat) | omnivore | plants, lichen and kelp, bird and fish parts, eggs  (Diets of wild *R. norvegicus* are very scantily documented; instead, the assumption that “rats are exceedingly versatile in their food habits [which] is one of the most familiar facts about them; it has obviously contributed to their success both as pests and as laboratory animals” [p. 44, 10] prevails.) | [11] |
| *Thomomys mazama*  (gopher) | herbivore | Grasses, forbs, woody plants, roots | [12] |

Appendix references

1. Gyug LW (2000) Status, distribution, and biology of the mountain beaver, *Aplodontia rufa*, in Canada. Canadian Field-Naturalist 114: 476-490.

2. Fedriani JM, Fuller TK, Sauvajot RM (2001) Does availability of anthropogenic food enhance densities of omnivorous mammals? An example with coyotes in southern California. Ecography 24: 325.

3. Quinn T (1997) Coyote (*Canis latrans*) food habits in three urban habitat types of western Washington. Northwest Science 71: 1-5.

4. Sovada MA, Telesco DJ, Roy CC (2000) Coyote, *Canis latrans*, use of commercial sunflower, *Helianthus* spp., seeds as a food source in western Kansas. Canadian Field-Naturalist 114: 697-699.

5. Asher M, De Oliveira ES, Sachser N (2004) Social system and spatial organization of wild guinea pigs (*Cavia aperea*) in a natural population. Journal of Mammalogy 85: 788-796.

6. Guichon ML, Cassini MH (1998) Role of diet selection in the use of habitat by pampas cavies *Cavia aperea* pamparum (Mammalia, Rodentia). Mammalia 62: 23-35.

7. Cornely JE, Verts BJ (1988) *Microtus townsendii*. Mammalian Species 325: 1-9.

8. Whitaker JO (1966) Food of *Mus musculus,* *Peromyscus maniculatus bairdi* and *Peromyscus leucopus* in Vigo County Indiana. Journal of Mammalogy 47: 473-486.

9. Kuijper DPJ, van Wieren SE, Bakker JP (2004) Digestive strategies in two sympatrically occurring lagomorphs. Journal of Zoology 264: 171-178.

10. Barnett SA (2007) The rat: a study in behavior. New Brunswick: Aldine Transaction. pp. 288.

11. Witmer G, Burke P, Jojola S, Dunlevy P (2006) The biology of introduced Norway rats on Kiska Island, Alaska, and an evaluation of an eradication approach. Northwest Science 80: 191-198.

12. Verts BJ, Carraway LN (2000) *Thomomys mazama*. Mammalian Species 641: 1-7.
